# A Fungal α-Galactosidase from *Tricholoma matsutake* with Broad Substrate Specificity and Good Hydrolytic Activity on Raffinose Family Oligosaccharides

**DOI:** 10.3390/molecules200813550

**Published:** 2015-07-24

**Authors:** Xueran Geng, Guoting Tian, Yongchang Zhao, Liyan Zhao, Hexiang Wang, Tzi Bun Ng

**Affiliations:** 1 State Key Laboratory for Agrobiotechnology, Department of Microbiology, China Agricultural University, Beijing 100193, China; E-Mail: gengxueran2007@163.com; 2 Institute of Biotechnology and Germplasmic Resource, Yunnan Academy of Agricultural Science, Kunming 650223, China; E-Mails:tiangt@aliyun.com (G.T.); yaasmushroom@aliyun.com (Y.Z.); 3 College of Food Science and Technology, Nanjing Agricultural University, Weigang, Nanjing 210095, China; E-Mail: zhlychen@njau.edu.cn; 4 School of Biomedical Sciences, Faculty of Medicine, The Chinese University of Hong Kong, Shatin, New Territories, Hong Kong, China

**Keywords:** mushroom, *Tricholoma matsutake*, álpha-galactosidase, characterization

## Abstract

An acidic α-galactosidase designated as TMG was purified from the fruiting bodies The purification protocol entailed ion exchange chromatography on Q-Sepharose and of *Tricholoma matsutake* with 136-fold purification and a specific activity of 909 units/mg. Mono-Q and fast protein liquid chromatography on Superdex 75. TMG is a monomeric protein exhibiting a molecular mass of 47 kDa in SDS-PAGE and gel filtration. The purified enzyme was identified by LC-MS/MS and three inner amino acid sequences were obtained. The optimum pH and temperature for TMG with pNPGal as substrate were pH 4.5 and 55 °C, respectively. The α-galactosidase activity was strongly inhibited by K^+^, Ca^2+^, Cd^2+^, Hg^2+^, Ag^+^ and Zn^2+^ ions. The enzyme activity was inhibited by the chemical modification agent N-bromosuccinimide (NBS), indicating the importance of tryptophan residue(s) at or near the active site. Besides hydrolyzing pNPGal, TMG also efficaciously catalyzed the degradation of natural substrates such as stachyose, raffinose, and melibiose. Thus TMG can be exploited commercially for improving the nutritional value of soy milk by degradation of indigestible oligosaccharides.

## 1. Introduction

α-Galactosidases (EC 3.2.1.22) are exo-glycosidases that catalyze the hydrolysis of the terminal α-linked galactoside residues from different substrates including melibiose, raffinose, stachyose, verbascose, as well as their derivatives [1]. Raffinose-family oligosaccharides, which are widely distributed in soy products, cannot be absorbed by human and other monogastirc animals leading to flatus formation [2]. Treatment with α-galactosidase for reducing stachyose and raffinose content in soybean flour has been investigated. The carbohydrase has been used in many industrial applications, mainly in the food and feed industries [3,4,5]. There are several industrial applications of α-galactosidases, mainly in the sugar industry, where they improve the crystallization of sucrose by hydrolyzing the raffinose in beet sugar syrups [6].

α-Galactosidases are widely distributed in microorganisms, plants, and animals. In recent years, many α-galactosidases have been extracted and purified from various sources. In plants, α-galactosidases are maximally distributed in seeds, fruits and leaves [7,8,9,10]. In microorganisms, α-galactosidases have been purified from the bacterium *Flavobacterium* sp. TN17 [11], fungi such as *Aspergillus terreus* [12], and the mushroom *Ganoderma lucidum* [13]. Some thermophilic enzymes have been discovered, such as a thermophilic α-galactosidase from *Neosarotrya fischeri* P1 [14], and thermostable α-galactosidases from *Lenzites elegans* [15], *Thielavia terrestris* NRRL8126 [16], and *Talaromyces emersonii* [17]. The thermostability makes them suitable candidates for use in feed industries.

*Tricholoma matsutake*, an ectomycorrhizal fungus, produces commercially important mushrooms that have iconic significance in the Far East and is highly valued as a delicacy and medicine. *T. matsutake* not only exhibits a delicate flavor, but also has diverse biological activities, such as immunomodulatory, anti-tumor and anti-oxidant activities [18,19,20]. Many bioactive substances have been purified from *T. matsutake*. A laccase from *T. matsutake* demonstrated activity in decolorization of azo dyes without a mediator [21]. A nuclease [22] and a polysaccharide [23] have been isolated from the fruiting bodies of *T. matsutake*. The enzyme and the polysaccharide are useful in the protection of the environment and human health. Now we detected high α-galactosidase activity in *T. matsutake*.

In the present study we reported, for the first time, the isolation and characterization of an α-galactosidase from *T. matsutake* named TMG. We also noted the effect of TMG on the degradation of raffinose oligosaccharides. This work would provide new evidence for further exploring the possibility of using TMG in industrial applications and advocating *T. matsutake* as a functional food.

## 2. Results and Discussion

### 2.1. Purification of TMG

As summarized in Table 1, the α-galactosidase from *T. matsutake* was purified 136-fold to homogeneity resulting in a final specific activity of 909 U/mg. The specific activity was 1.6-fold of that (561 U/mg) of α-galactosidase from a fan-shaped mushroom *Coriolus versicolor* [24]. The purification protocol for TMG consisted of ion exchange chromatography on Q-Sepharose and Mono-Q and gel filtration on Superdex 75 HR. Activity was located successively in peaks Q2, MonoQ1 and SU1.

**Table 1:** Purification of *Tricholoma matsutake* α-galactosidase.

Chromatographic Fraction	Total Protein (mg/400 g)	Total Activity (U) ^a^	Specific Activity (U/mg) ^b^	Yield (%)	Purification Fold ^c^
Crude extract	17,427	116,204	6.7	100	1
Q2	311.7	102,037	327.3	87.9	49.1
MonoQ1	95.6	34,490	360.8	29.7	54.1
SU2	9.6	8727	909.1	7.5	136.3

**^a^** Total activity: α-galactosidase activity (U/mL) in each step × Volume (mL); ^b^ Specific activity: total activity/Total protein; ^c^ Purification fold: specific activity of each step/specific activity of the first step.

### 2.2. Determination of Molecular Weight and Amino Acid Sequence of TMG

**Figure 1:**
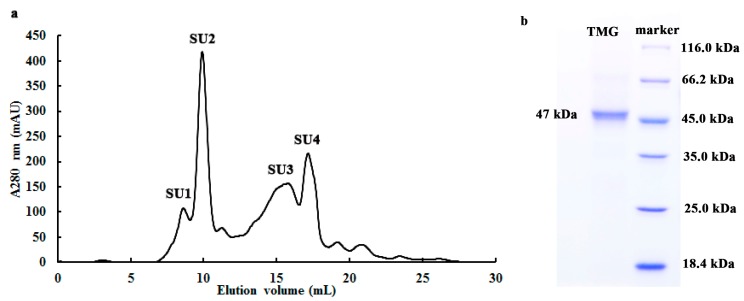
(**a**) FPLC-gel filtration on Superdex 75 10/300 GL column and (**b**) SDS-PAGE of fraction SU2. (**a**) Eluent: 10 mM NaOAC-HOAC buffer (pH 5.2). Fraction size: 0.8 mL; Flow rate: 0.5 mL·min^−1^. Fraction SU1 represents purified *Tricholoma matsutake* α-galactosidase; (**b**) SDS-PAGE of fraction SU2.

Purified TMG was enriched in a symmetrical peak SU2 (with a molecular mass of 47 kDa, Figure 1a) and appeared as a single band with a molecular mass of 47 kDa in SDS-PAGE (Figure 1b). Hence the molecular mass of TMG was judged to be 47 kDa. Previously only a few α-galactosidases were purified from mushrooms, including *Coriolus versicolor*, *Ganoderma lucidum*, *Lenzites elegans* and *Pleurotus florida* and their molecular masses are spread over a wide range from 70 kDa to 249 kDa [13,15,24,25]. Hence α-galactosidases from different sources display different molecular masses and subunit properties. Although the molecular mass of TMG was lower than counterparts purified from other mushrooms, it was similar to PGG purified from *Phaseolus coccineus* (43 kDa) [10] and the α-galactosidase from *Cicer arietinum* (45 kDa) [26].

The inner amino acid sequences of TMG obtained by LC-MS/MS and database search using BLAST indicated that the peptides showed some similarity with α-galactosidases from other sources. Peptide GNVMVSLG exhibited 88% identity to α-galactosidases from *Bipolaris maydis* C5 (accession number EMD92465.1), *Bipolaris victoriae* F13 (accession number EUN27106.1), *Bipolaris oryzae* ATCC 44560 (accession number XP_007687294) and *Bipolaris sorokiniana* ND90Pr (accession number XP_007697049.1), which all belong to the glycosyl hydrolase 27 family. Peptide LLNMND showed 83% identity with the α-galactosidase from *Lactobacillus otakiensis* (accession number GAD17169.1) and 75% identity with α-galactosidase from *Kutzneria* sp. 744 (accession number WP_043715631.1). Peptide INVNDS manifested 100% identity with *Bacteroides nordii* α-galactosidase (accession number WP_025867713).

### 2.3. Biochemical Properties of TMG

**Figure 2:**
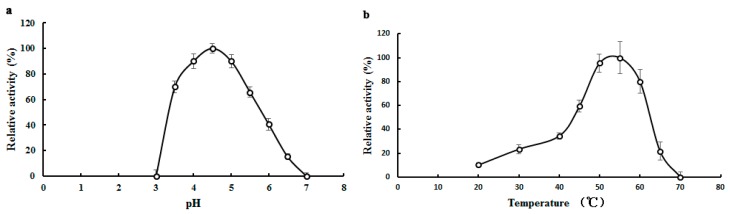
Effects of pH and temperature on the activity of *Tricholoma matsutake* α-galactosidase. (**a**) Effect of pH on TMG. Buffer: 0.1 M Na_2_HPO_4_-citric acid buffers. Results represent mean ± SD (*n* = 3); (**b**) Effect of temperature on TMG. Results represent mean ± SD (*n* = 3).

With pNPGal as substrate, the optimal pH value of TMG was observed at pH 4.5 (Figure 2a), which was identical with the optimal pH for α-galactosidases from *Neosartorya fischeri* [14]. *Lenzites elegans* [15] and *Talaromyces emersonii* [17]. The optimal pH values for α-galactosidase from *Aspergillus terreus* and an acidophilic fungus *Bispora* sp. MEY-1 were pH 5.5 and pH 3.5, respectively [12,27]. Although most of the optimal pH values for the α-galactosidases were an acidic pH, the optimal pH of α-galactosidase purified from *Bacillus megaterium* was 7.5 [28]. As shown in Figure 2a, when the pH was increased to 6, only 40.6% of the activity of TMG remained. When the pH was outside the range of 3.0 to 7.0, hardly any α-galactosidase activity could be detected.

α-Galactosidases purified from different sources exhibited good enzymatic activity over a wide range of temperatures. The enzyme from *Lenzites elegans* [15] showed a high level of activity at temperatures ranging from 60 to 80 °C. Maximal activity of TMG was observed at 55 °C (Figure 2b) which was in good agreement with the enzyme from *Bacillus megaterium* [28]. When treated at 40 °C for 2 h, the activity of TMG was completely preserved (data not shown). However, TMG showed poor thermostability at higher temperatures and was completely inactivated after incubation at 50 °C for 30 min.

**Figure 3:**
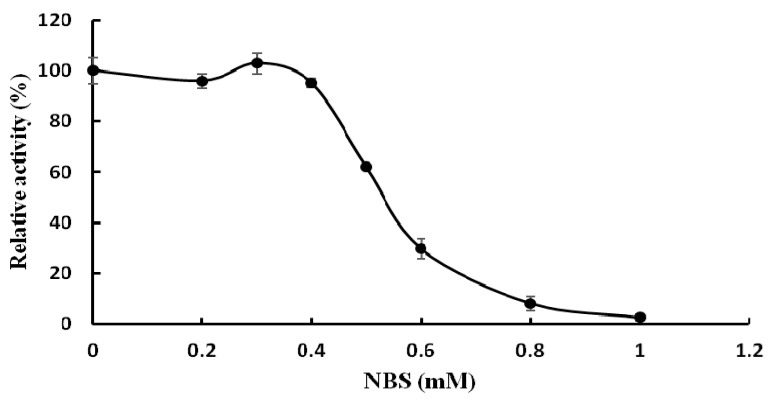
Effect of the chemical modification reagent *N*-bromosuccinimide (NBS) on activity of *Tricholoma matsutake* α-galactosidase (results represent mean ± SD, *n* = 3).

The effects of various metal ions and other chemicals on TMG activity were tested and TMG was found to be very sensitive to metal ions. The activity of TMG almost disappeared in the presence of K^+^, Ca^2+^, Cd^2+^, Hg^2+^, Ag^+^, and Zn^2+^ ions (1.25 mM–10 mM) and was strongly affected by Fe^3+^ ions (1.25 mM–10 mM) and Cu^2+^ ions (2.5 mM–10 mM). The total inhibition of α-galactosidase activity by Hg^2+^ and Ag^+^ ions was also seen in α-galactosidases from other sources, for example, the enzymes from *Cicer arietinum* [26] and *Tachigali multijuga* seeds [29]. It was reported by Singh [26] that the total inhibition by Ag^+^ ions was probably due to reaction of Ag^+^ ions with carboxyl and/or histidine residues. Hg^2+^ ions can react with thiol groups, amino and imidazolium groups of histidine and with peptide linkages, which was the reason why TMG was completely inhibited by Hg^2+^ ions [30]. Increasing the concentration of Mn^2+^, Mg^2+^, Pb^2+^, Al^3+^ ions (1.25 mM–10 mM) had little or no effect on TMG activity (Table 2). The activity of TMG was not inhibited by EDTA, implying that the enzyme is not a metalloenzyme or does not require divalent cations for activity. Similar to the present findings, EDTA had no effect on the activity of *Bacillus megaterium* and *Ganoderma lucidum* α-galactosidases [13,28]. While EDTA slightly inhibited the α-galactosidase activity from *Lenzites elegans* [15]. The chemical reagents modified different amino acid functional groups. The amino group of tryptophan was chemically modified by NBS while the histidine residues were modified by diethylpyrocarbonate (DEPC) [31]. The carboxyl groups were modified by carbodiimide (EDC) [32]. The disulfide bond and the arginine residues were chemically modified by dithiothreitol (DTT) [33] and diacetyl (DIC), respectively. The chemical modification reagent NBS drastically inhibited the activity of TMG. Incubation of TMG with 1 mM NBS for 30 min resulted in complete loss of α-galactosidase activity (Figure 3), which indicated the pivotal role of tryptophan at or near the active site. This finding was in keeping with the results reported for the α-galactosidases purified from *Coriolus versicolor* [24] and *Phaseolus coccineus* seeds [10]. Other chemical modification reagents (DEPC, EDC, DIC, DTT) had virtually no effect on TMG (data not shown), which indicated that the groups of tryptophan, histidine, carboxyl, arginine residues and disulfide bonds were not present at or in the proximity of the active site.

**Table 2:** Effects of different metal ions on activity of *Tricholoma matsutake* α-galactosidase ^a^.

Metal Ion Concentration	Relative—Galactosidase Activity (%)			
	10 mM	5 mM	2.5 mM	1.25 mM
Mg^2+^	30.6 ± 0.03	80.90.01	82.6 ± 0.02	93.0 ± 0.04
Mn^2+^	43.3 ± 0.03	69.1 ± 0.07	101.6 ± 0.02	100.4 ± 0.02
Pb^2+^	9.6 ± 0.02	80.0 ± 0.12	88.3 ± 0.09	95.3 ± 0.05
Al^3+^	72.1 ± 0.01	80.7 ± 0.03	73.1 ± 0.05	82.5 ± 0.02
Cu^2+^	ND	ND	31.8 ± 0.01	80.9 ± 0.05
Fe^3+^	ND	ND	ND	31.4 ± 0.02
EDTA	116.4 ± 0.65	96.3 ± 0.84	101.3 ± 0.61	112.5 ± 0.45

^a^ The activity of α-galactosidase without incubation with metal ions was set as 100%. Results represent mean ± SD, *n* = 3. The detailed data for each metal ion (Mg^2+^, Mn^2+^, Pb^2+^, Al^3+^, Cu^2+^ and Fe^3+^) are shown in Tables S1–S6 in the supplementary file. “ND” means not detected.

### 2.4. Substrate Specificity of TMG and Determination of Kinetic Parameters

The purified TMG showed different hydrolytic activities towards various natural and synthetic substrates (Table 3). The highest activity was demonstrated toward pNPGal, which was much higher than the activities toward other nitrophenyl derivatives (oPNPGal, 4-nitrophenyl β-d-glucuronide). TMG had hardly any activity on synthetic substrates oPNPGal, and 4-nitrophenyl β-d-glucuronide compared with pNPGal. This suggests that probably the para configuration of the substrate pNPGal facilitated its access to the active site of the enzyme. In this study, TMG exhibited higher activity toward natural substrates (stachyose, raffinose and melibiose) compared to synthetic nitrophenyl derivatives (oPNPGal, 4-nitrophenyl β-d-glucuronide). The rate of hydrolysis of natural substrates was in the order raffinose (84%) > stachyose (67%) > melibiose (17%). In particular, the relative activity of TMG for raffinose attained 84%. TMG showed meager activity on the polysaccharides, locust bean gum and guar gum, with the rate of hydrolysis being 12% and 13% respectively.

**Table 3:** Hydrolysis of different substrates by *Tricholoma matsutake* α-galactosidase.

Substrate	Concentration (mM)	Relative Activity (%) ^a^
4-Nitrophenyl α-d-galactopyranoside (pNPG)	10	100 ± 0.77
2-Nitrophenyl β-d-galactopyranoside (oNPG)	10	0.9 ± 0.03
4-Nitrophenyl β- d-glucuronide	10	4 ± 0.01
Stachyose	50	67 ± 0.02
Raffinose	50	84 ± 0.01
Melibiose	50	17 ± 0.11
Locust bean gum	1%	12 ± 0.07
Guar gum	1%	13 ± 0.05

^a^ Relative activities were calculated in comparison with pNPGal activity, which was considered as 100%. Results represent mean ± SD, *n* = 3.

The K_m_ values of TMG for hydrolysis of pNPGal, raffinose and stachyose were 0.99 mM, 3.7 mM and 3.5 mM, respectively. This was in keeping with the above results showing that TMG displayed a higher affinity (lower Km) toward pNPGal than natural oligosaccharides as substrates. The calculated K_m_ of TMG was lower than that of rGalQ17 (K_m_ = 2.25 mM) from *Flavobacterium* ap. TN17a [11], and a little higher than that of the α-galactosidase (K_m_ = 0.7 mM) from *Cicer arietinum* [26], but much higher than that of the α-galactosidase from *Coriolus versicolor* [24]. The catalytic efficiency expressed by K_cat_/K_m_ showed that the substrate pNPGal was used most efficiently by the enzyme (Table 4).

**Table 4:** Kinetic parameters for hydrolysis of pNPGal, raffinose and stachyose by *Tricholoma matsutake* α-galactosidase.

Substrate	K_m_ (Mm)	K_cat_^a^ (s^−1^)	K_cat_/K_m_ (s^−1^·mM^−1^)
pPNGal	0.99	9.17	9.3
Raffinose	3.7	6.4	1.73
Stachyose	3.5	6.7	1.9

^a^ Turnover number (K_cat_) is defined here as the number of mmoles of product s^−1^·mmol^−1^ of α-galactosidase, given by the relationship V_max_/[E].

**Figure 4:**
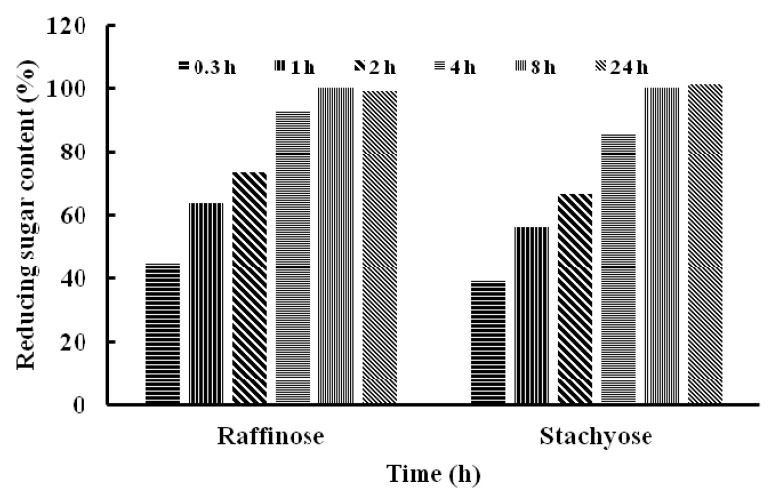
The content of reducing sugar after hydrolysis of raffinose and stachyose by *Tricholoma matsutake* α-galactosidase.

Stachyose and raffinose were digested by TMG for 0.3, 1, 2, 4, 8 and 24 h, respectively. The content of reducing sugar increased gradually after treatment with TMG in the time-course of hydrolysis, which indicated that stachyose and raffinose were hydrolyzed effectively to yield galactose and sucrose (Figure 4).

After 24 h, the content of reducing sugar released by the two oligosaccharides did not show a further increase compared with that at 8 h. The difference in efficiency of hydrolysis of the two oligosaccharides catalyzed by TMG was not remarkable, which was consistent with the K_m_ of TMG to stachyose (K_m_ = 3.5 mM) and raffinose (K_m_ = 3.7 mM). Because humans and monogastric animals lack the enzyme α-galactosidase, raffinose family oligosaccharides, mainly stachyose and raffinose, cannot be absorbed and accumulate resulting in their microbial fermentation and flatus formation [2]. It is possible to improve the nutritional value of soymilk by reducing the content of RFOs and exogenous enzyme treated with RFOs has been proven to be a more effective and reliable method [10]. The ability of TMG to hydrolyze RFOs make it a good candidate as an additive for biotechnological applications. Besides, *T. matsutake* is a well-known wild edible mushroom and this finding can prompt further exploitations of the functions and activities of *T. matsutake*. There are also reports on α-galactosidases from other sources which degrade RFOs. Similarly, an α-galactosidase from symbiotic *Flavobacterium* sp. TN17 efficiently hydrolyzed many natural substrates including stachyose, raffinose and melibiose [11]. Wang *et al*., revealed that after treatment with one of α-galactosidases from *Neosartorya fischeri* P1 at 50 °C for 3 h, the raffinose and stachyose contents were decreased by 69.9% and 94.5%, respectively [14].To recapitulate, we have reported the purification and characterization of an α-galactosidase from *T. matsutake*. The good capacity of TMG in the degradation of raffinose oligosaccharides was noteworthy. Though the wild edible mushroom *T. matsutake* is highly valued as a delicacy and medicine, it is wild and cannot be cultivated. In order to have better productivity and facilitate the enzyme purification, it is necessary to clone the enzyme in the near future. Many α-galactosidases were cloned and a high-level expression of α-galactosidase from *Rhizomucor miehei* [34], the recombinant enzyme from *Flavobacterium* sp TN17 [11] and an α-galactosidase from *Neosartorya fischeri* P1 with significant hydrolysis ability of milk [14] could easily be obtained, TMG showed poor thermostability, it can be improved by immobilizing it onto functionalized graphene or onto chitosan and Amberlite, which were based on the immobilization of *Cicer* α-galactosidases [35,36]. The three immobilized *Cicer* α-galactosidases had higher thermal stability than the soluble enzymes and effective hydrolysis of RFOs increases the prospects of application of *Cicer* α-galactosidases in food processing industries. The TMG can also be non-covalently immobilized on a reversibly soluble-insoluble polymer [37] or immobilized on Sepabesd EC-EA and Sepabead EC-HA [38].

In future investigations, combing the cloning of the enzyme to produce higher enzyme amounts easier to be purified [11,14,34] and a suitable enzyme immobilization to improve the enzyme features (stability, activity, selectivity) [39,40,41] may convert this promising enzyme into a suitable product with potential applications in industry.

## 3. Experimental Section

### 3.1. Materials

*Tricholoma matsutake* fruiting bodies were collected from Yunnan Province (China). Q-Sepharose, Mono-Q, Superdex 75 HR 10/30 and AKTA Purifier were purchased from GE Healthcare (Uppsala, Sweden). The substrates, 4-nitrophenyl-α-D-galactophyranoside (pNPGal), 2-nitrophenyl β-d-galactopyranoside (oNPGal), 4-nitrophenyl β-d-glucuronide, locust bean gum, guar gum, melibiose, stachyose and raffinose were purchased from Sigma Chemical Company (St. Louis, MO, USA). All other chemicals used were of analytical grade unless otherwise stated.

### 3.2. Enzyme Activity Assay

The activity of α-galactosidase was assayed with the pNPGal method as described [42] with modification. Diluted enzyme (50 μL) was incubated with 50 μL 10 mM pNPGal (pH 4.6) at 50 °C for 10 min. The reaction was terminated by adding 400 μL 0.5 M Na_2_CO_3_ and the released *p*-nitrophenol was determined spectrophotometrically at 405 nm. One unit of α-galactosidase activity was defined as the amount of enzyme required to release 1 μmol of *p*-nitrophenol per min at 50 °C and pH 4.6.

### 3.3. Purification of α-Galactosidase from Tricholoma Matsutake

Fractionation of an extract of *Tricholoma matsutake* fresh fruiting bodies (400 g) was carried out by ion exchange chromatography using a 10 cm × 100 cm column of Q-Sepharose. The extract was homogenized in distilled water (1:2, *w*:*v*) with a Waring blender followed by extraction overnight at 4 °C. Then the homogenate was centrifuged at 9000 rpm for 20 min at 4 °C. The supernatant was collected and applied to a Q-Sepharose column in 10 mM NaOAc-HOAc buffer (pH 5.2). After removal of the unadsorbed fraction Q1, adsorbed proteins were eluted stepwise with 100 mM NaCl in NaAc-HAc buffer to yield fraction Q2 and then eluted with 1.0 M NaCl in NaOAc-HOAc buffer to yield fraction Q3. α-Galactosidase activity was located in fraction Q2. After dialysis against distilled water, fraction Q2 was subjected to ion exchange chromatography on Mono-Q. After the unadsorbed fraction MQ1 was eluted, the column was further eluted with a linear 0–1.0 M NaCl gradient in the same NaOAc-HOAc buffer (pH 5.6). The active fraction MQ1 was further purified by gel filtraction on a fast protein liquid chromatography Superdex 75 10/300 GL column in 10 mM NaOAc-HOAc buffer (pH 5.2).

### 3.4. Determination of Molecular Weight and Amino Acid Sequence

The purified α-galactosidase from *Tricholoma matsutake* was subjected to SDS-PAGE [43] to assesss protein homogeneity and to determine molecular mass. The native molecular mass of the α-galactosidase was determined with size exclusion chromatography using a Superdex 75 column. The α-galactosidase band obtained after SDS-PAGE was destained, digested with trypsin, and then dissolved in 0.1% formic acid and 2% acetonitrile for liquid chromatography-tandem mass spectrometry (LC-MS/MS) analysis using an LTQ—Orbitrap mass spectrometer (Thermo Electron, Bremen, Germany).

### 3.5. Biochemical Properties of the Enzyme

In the assay for optimum pH, a solution of 10 mM pNPGal, which was used as substrate, was freshly prepared in the pH range from pH 2.0–8.0 using 100 mM Na_2_HPO_4_-citric acid buffer, and the activity of the α-galactosidase from *Tricholoma matsutake* was recorded at each pH value to ascertain the pH associated with the maximal activity. For determining the optimum temperature, the activity of the α-galactosidase toward the substrate pNPGal was assayed over the temperature range of 20–70 °C at the optimum pH. The effects of various metal ions and chemical reagents(Na^+^, K^+^, Al^3+^, Zn^2+^, Mg^2+^, Mn^2+^, Fe^3+^, Ca^2+^, Cu^2+^, Cd^2+^, Hg^2+^, Pb^2+^, EDTA, NBS, DTT, DIC, EDC, DEPC) on the activity of purified enzyme were examined. The enzyme was incubated with a series of concentrations of metal ions and chemical reagents at 4 °C for 2 h and the remaining activity toward pNPGal was assayed as described above.

### 3.6. Substrate Specificity and Kinetic Parameters

In order to determine the substrate specificity of the purified α-galactosidase, various synthetic and natural substrates were used in the assay. The activity against synthetic substrates, pNPGal, oNPGal, 4-nitrophenyl β-d-glucuronide (10 mM), was determined under standard conditions as described for pNPGal. The ability to hydrolyze natural substrates (locust bean gum, guar gum, melibiose, stachyose and raffinose) was determined by measuring the reducing sugar released using 3,5-dinitrosalicylic acid as described by Miller [44]with slight modification. The reaction mixture consisting of appropriately diluted enzyme and 10 mM sugar in 100 mM NaAc-HAc buffer (pH 4.6) was incubated at 40 °C for 30 min. One unit of α-galactosidase activity was defined as the amount of enzyme required to release 1 μmol of reducing sugar equivalent to galactose per minute under the assay conditions. While the glucose released by the substrate melibiose was measured with a glucose-oxidase kit (Beijing BHKT Clinical Reagent Co., Ltd. Beijing, China). One unit of the enzyme activity was defined as the amount of enzyme required to release 1μmol of glucose per minute at 37 °C. The kinetic parameter K_m_ of the purified α-galactosidase on different substrates was determined. pNPGal (1–10 mM), stachyose(1–10 mM) and raffinose(1–10 mM) were used as substrates, and the reactions were performed in the same conditions as before. The apparent Michaelis constant K_m_ was calculated using the Grafit program.

## Supplementary Materials

Supplementary materials can be accessed at: http://www.mdpi.com/ 1420-3049/20/08/13550/s1.
