# Supplementary material for: A Fungal α-Galactosidase from Tricholoma matsutake with Broad Substrate Specificity and Good Hydrolytic Activity on Raffinose Family Oligosaccharides
Source: Molecules. 2015 Jul 24;20(8):13550–62. doi: 10.3390/molecules200813550 (PMC6332393; doi:10.3390/molecules200813550)
Supplement: Supplementary file 1 [file molecules-20-13550-s001.pdf]

Supplemental Material

Table S1. Effects of Mg^{2+} ions on activity of *Tricholoma matsutake* α-galactosidase.

Concentration (mM)	CK	OD405	Average of OD405	0D405-CK	Realtive Activity (%)
		0.223			
10	0.094	0.167	0.200333	0.106333	30.6 ± 0.03
		0.211			
		0.382			
5	0.089	0.355	0.370333	0.281333	80.9 ± 0.01
		0.374			
		0.372			
2.5	0.086	0.392	0.373333	0.287333	82.6 ± 0.02
		0.356			
		0.448			
1.25	0.084	0.398	0.407333	0.323333	93.0 ± 0.04
		0.376			

The activity of α-galactosidase without incubation with metal ions was set as 100%, it was 0.34767.

Table S2. Effects of Mn^{2+} ions on activity of *Tricholoma matsutake* α-galactosidase.

Concentration (mM)	CK	OD405	Average of OD405	0D405-CK	Realtive Activity (%)
		0.230			
10	0.084	0.207	0.234667	0.150667	43.3 ± 0.03
		0.267			
		0.365			
5	0.082	0.362	0.322333	0.240333	69.1 ± 0.07
		0.24			
		0.412			
2.5	0.082	0.436	0.435333	0.35333	101.6 ± 0.02
		0.458			
		0.455			
1.25	0.1	0.431	0.449	0.349	100.4 ± 0.02
		0.461			

The activity of α-galactosidase without incubation with metal ions was set as 100%, it was 0.34767.

Table S3. Effects of Pb^{2+} ions on activity of *Tricholoma matsutake* α-galactosidase.

Concentration (mM)	CK	OD405	Average of OD405	0D405-CK	Realtive Activity (%)
		0.152			
10	0.1	0.134	0.13333	0.0333	9.6 ± 0.02
		0.114			
		0.236			
5	0.91	0.411	0.369	0.278	80.0 ± 0.12
		0.460			
		0.301			
2.5	0.98	0.441	0.405	0.307	88.3 ± 0.09
		0.473			
		0.460			
1.25	0.083	0.427	0.414333	0.33133	95.3 ± 0.05
		0.356			

The activity of α-galactosidase without incubation with metal ions was set as 100%, it was 0.34767.

Table S4. Effects of Al^{3+} ions on activity of *Tricholoma matsutake* α-galactosidase.

Concentration (mM)	CK	OD405	Average of OD405	0D405-CK	Realtive Activity (%)
10	0.076	0.332<br>0.327<br>0.321	0.320667	0.250667	72.1 ± 0.01
5	0.086	0.340<br>0.398<br>0.362	0.280667	0.280667	80.7 ± 0.03
2.5	0.072	0.267<br>0.354<br>0.357	0.254	0.254	73.1 ± 0.05
1.25	0.074	0.380<br>0.334<br>0.368	0.286667	0.286667	82.5 ± 0.02

The activity of α-galactosidase without incubation with metal ions was set as 100%, it was 0.34767.

Table S5. Effects of Cu^{2+} ions on activity of *Tricholoma matsutake* α-galactosidase.

Concentration (mM)	CK	OD405	Average of OD405	0D405-CK	Realtive Activity (%)
10	0.515	0.147<br>0.139<br>0.138	0.141333	−0.37367	ND
5	0.267	0.149<br>0.138<br>0.139	0.142	−0.125	ND
2.5	0.123	0.270<br>0.146<br>0.258	0.233667	0.110667	31.8 ± 0.01
1.25	0.118	0.390<br>0.373<br>0.435	0.399333	0.281333	80.9 ± 0.05

The activity of α-galactosidase without incubation with metal ions was set as 100%, it was 0.34767.

Table S6. Effects of Fe^{3+} ions on activity of *Tricholoma matsutake* α-galactosidase.

Concentration (mM)	CK	OD405	Average of OD405	0D405-CK	Realtive Activity (%)
10	1.198	0.787<br>0.807<br>0.795	0.796333	−0.40167	ND
5	0.678	0.452<br>0.341<br>0.411	0.401333	−0.27667	ND
2.5	0.370	0.350<br>0.288<br>0.324	0.320667	−0.04933	ND
1.25	0.224	0.345<br>0.348<br>0.307	0.333333	0.190333	31.4 ± 0.02

The activity of α-galactosidase without incubation with metal ions was set as 100%, it was 0.34767.
